# Workforce Science and the Human–AI economy of health and care

**DOI:** 10.1371/journal.pgph.0007331

**Published:** 2026-09-21

**Authors:** Jim Campbell

**Affiliations:** King’s Population Health Institute, King’s College London, London, United Kingdom; PLOS: Public Library of Science, UNITED STATES OF AMERICA

Health and care systems are undergoing profound transformation. Population ageing, epidemiological transition, widening inequities, changing expectations of citizens and rapid advances in artificial intelligence (AI) are reshaping how health and care are organised, delivered and governed. Increasingly, health and wellbeing are co-produced through the combined capabilities of people and intelligent technologies operating across homes, communities, primary care, hospitals and virtual environments. Yet the scientific frameworks used to analyse and steward the health and care workforce remain largely rooted in models developed for a different era.

This Opinion argues that health workforce development and management is fundamentally about stewardship and how public policy governs the complex relationships between people, organisations and technologies to achieve equity in population health. It encourages governments and all relevant stakeholders to reimagine the health and care workforce as a strategic capability asset and proposes Workforce Science as a disciplined science of measuring, analysing and stewarding this capability in the Human–AI Economy of Health and Care.

This conceptual shift reflects the evolution of health workforce policy and economic thinking since 2000. The *World Health Report 2006* [1] and the establishment of the Global Health Workforce Alliance elevated the health workforce from a national ‘manpower’ or human resource concern to a global policy priority. A decade later, WHO's *Global Strategy on Human Resources for Health: Workforce 2030* and its promotion of health labour market analysis established a more rigorous understanding of how the workforce is educated, financed and employed [2]. Labour market analysis transformed workforce policy by demonstrating that labour supply and demand, rather than population need alone, shape workforce availability and performance [3,4]. In parallel, the evolution in labour economics has given greater emphasis to human benefits and outcomes. Becker’s original conceptualisation of labour as *human capital* had an emphasis on productivity and earnings [5]. Entering the new millennium, Sen reframed economic development around *human capability* and the freedoms that enable people to lead lives they have reason to value [6], while Nussbaum further developed capability as the basis of *human flourishing* [7]. More recently, Bueno has argued that systems should be organised around the *human economy*, in which value derives not simply from productive activity but from contributing to the wellbeing of others and society [8–10]: a concept particularly resonant for jobs in the health and care ecosystem.

The evolution in ideas reframes how to envision the purpose and stewardship of ‘labour’, ‘work’ and ‘workers’. When applied to ISIC Sector Q – Human Health and Social Work Activities [11], labour in this sector – predominantly female - has far greater added value for societal progress and national wellbeing frameworks [12]. And as AI becomes increasingly embedded within ‘Sector Q’, the conceptual progression advances to a ‘human–AI economy’ and how this hybrid capability asset contributes to human flourishing and wellbeing for the health worker, the patient and the population.

Workforce Science builds directly upon these foundations, recognising that the object of enquiry has fundamentally changed: capability is no longer solely human. It increasingly emerges through the complex interactions of workers, workforce teams and intelligent technologies. This complexity should not be reduced to a debate on the automation of tasks and the consequent substitution or replacement of humans with AI. Instead, it demands a higher degree of scientific enquiry and in the health setting, greater attention to epistemic resilience [13]. The stewardship challenge pivots from supply and demand questions on “How many workers do I need to educate and employ?” to “How is hybrid human-AI capability created, distributed, and stewarded to advance health and wellbeing?”

Workforce Science provides the scientific discipline within which complementary approaches on AI, demography, epidemiology, labour economics, statistical systems and workforce planning can be integrated to understand, measure and steward this national capability asset. The conceptual construct of effective capability (C*) is understood as a function of education financing (E*f*), education quality (E*q*), labour (L), the Human–AI dynamic (H-AI), governance capacity (G), and the conditions, rights and realities of work (R) (see Table 1).

**Table 1:** Effective capability (C*).

Symbol	Definition
C*	**Effective capability**, denoting the workforce capability in the Human–AI economy of health and care, distinguishing it from labour-based or education-based measures of capacity.
**E**	**Education** within Workforce Science encompasses the formation, maintenance and adaptation of capability across the workforce lifecycle, including undergraduate and postgraduate education, technical and vocational education and training, apprenticeships, workplace learning, continuing development, upskilling and reskilling.
**E*f***	**Education *financing***, denoting the allocation, management, and reporting of financial resources invested in health workforce education and training and throughout the workforce lifecycle, including the capacity to track public, private and out-of-pocket flows across the education and health sectors over time.
**E*q***	**Education *quality***, denoting the standards, relevance, performance, and outcomes of the many education, learning and training pathways that determine workforce capability, competence and preparedness for contemporary and future practice as individuals and teams.
**L**	**Labour**, denoting the availability, participation, deployment, and productivity of the workforce across the Spectrum of Work (paid/unpaid; formal/informal) in Sector Q and beyond.
**H–AI**	**Human–AI dynamic**, denoting the capabilities emerging through the complementary interaction of workers (individuals and teams) and artificial intelligence in both the education and training of the workforce and the delivery, management, and improvement of population health and care services.
**G**	**Governance**, denoting the stewardship, regulation, financing, accountability, and institutional arrangements that enable or limit workforce capability and system performance.
**R**	**Conditions, rights and realities of work**, denoting the protection and fulfilment of the rights, responsibilities, safety, dignity, and wellbeing of workers as foundational conditions for sustainable capability and human flourishing.

Labour remains fundamental but capability is distributed across professionals, patients, carers and AI-enabled systems and can no longer be proxied by employment of licensed health professionals, their full-time equivalents, or density per 10,000 population. Instead, it expands on the logic in earlier workforce planning models examining the variation in the availability, accessibility, acceptability and quality of human resources for health [14] by introducing analytical layers on human-AI capability, governance and decision integrity, and the conditions of work.

To ensure rigour in the analysis and stewardship of this capability asset, Workforce Science progresses through five layers that mirror the logic of scientific enquiry (see Table 2). It first defines what exists, then determines what is measured, how evidence is analysed, how evidence informs decisions, and finally how those decisions are translated into practice. This progression of definition → measurement → analysis → governance → stewardship distinguishes Workforce Science from traditional workforce approaches by making lexical, ontological, numerical, analytical and decision disciplines explicit and cumulative [15]. The same flow enables structured appraisal of evidence in the published and grey literature and has additional utility for editorial boards of health journals and prospective academic commissions and government reviews on how AI will impact the future health and care workforce.

**Table 2:** Workforce science: Progressive layers from Definition → Measurement → Analysis → Governance → Stewardship.

Layer	Elements
**1. Lexical and Ontological Discipline**	the conceptual foundations of Workforce Science through the precise use and definition of language, occupations, classifications and relationships that establish the object of scientific enquiry and enable valid comparison within and across jurisdictions.
**2. Numerical Discipline**	the identification, measurement, accounting and contextualisation of the ‘Workforce of Interest’ – namely the explicitly defined population of workers, contributors and capability assets to which a workforce analysis, estimate or stewardship decision is intended to apply - through rigorous specification of numerators, denominators, measurement units, appropriate reference populations and associated costs.
**3. Analytical Discipline**	the selection and application of analytical methods and fit-for-purpose maturity models that ensure evidence is valid and proportionate for understanding workforce capability, distribution, equity, investment, productivity and change over time.
**4. Governance and Decision Integrity**	the transparency of the evidence-to-decision pathway through explicit assumptions, accountable interpretation and reproducible reasoning to generate trust in scientific, managerial and policy decisions.
**5. Political Economy and Stewardship**	the stewardship of human-AI capability as a strategic asset that aligns workforce capability with human flourishing, societal wellbeing, economic development, rights, ethics and long-term public value.

The rationale for Workforce Science is practical. Health and care workers have become central to economic prosperity, social structures and health security. In many OECD settings employment in Sector Q represents at least 1 in every 10 jobs, with Scotland recording it as 1 in 6 jobs and the UK identifying it as the fastest growing job sector in the last decade. In parallel, AI is rapidly becoming embedded in the co-production of health and care with enormous potential to augment human capability if managed appropriately. While existing workforce and research frameworks remain indispensable they were developed primarily to understand units of labour within analogue health systems rather than hybrid capability within a Human–AI Economy of Health and Care.

Scientific disciplines evolve when their object of enquiry changes. Workforce Science is proposed as a next stage in the evolution of the development and management of human resources for health, providing the lexical, ontological and numerical clarity, measurement science and architecture required to assist governments and stakeholders to steward one of their most strategic capability assets and to accelerate population health, wellbeing and human flourishing.
